# Translational PK-PD modeling analysis of MCLA-128, a HER2/HER3 bispecific monoclonal antibody, to predict clinical efficacious exposure and dose

**DOI:** 10.1007/s10637-018-0593-x

**Published:** 2018-05-05

**Authors:** Aurelia H. M. de Vries Schultink, Robert P. Doornbos, Alexander B. H. Bakker, Kees Bol, Mark Throsby, Cecile Geuijen, David Maussang, Jan H. M. Schellens, Jos H. Beijnen, Alwin D. R. Huitema

**Affiliations:** 1 Department of Pharmacy & Pharmacology, Antoni van Leeuwenhoek – The Netherlands Cancer Institute and MC Slotervaart, Louwesweg 6, 1066 EC Amsterdam, the Netherlands; 2 Merus N.V, Yalelaan 62, 3584 CM Utrecht, the Netherlands; 3 Department of Clinical Pharmacology, Antoni van Leeuwenhoek – The Netherlands Cancer Institute, P.O Box 90203, 1006 BE Amsterdam, the Netherlands; 4 Science Faculty, Utrecht Institute for Pharmaceutical Sciences (UIPS), Division of Pharmacoepidemiology & Clinical Pharmacology, Utrecht University, P.O. Box 80082, 3508 TB Utrecht, the Netherlands; 5 Department of Clinical Pharmacy, University Medical Center Utrecht, P.O. Box 85500, 3508 GA Utrecht, the Netherlands

**Keywords:** MCLA-128, Bispecific, Preclinical, Translational, PK-PD modeling

## Abstract

*Introduction* MCLA-128 is a bispecific monoclonal antibody targeting the HER2 and HER3 receptors. Pharmacokinetics (PK) and pharmacodynamics (PD) of MCLA-128 have been evaluated in preclinical studies in cynomolgus monkeys and mice. The aim of this study was to characterize the PK and PD of MCLA-128 and to predict a safe starting dose and efficacious clinical dose for the First-In-Human study. *Methods* A PK-PD model was developed based on PK data from cynomolgus monkeys and tumor growth data from a mouse JIMT-1 xenograft model. Allometric scaling was used to scale PK parameters between species. Simulations were performed to predict the safe and efficacious clinical dose, based on AUCs, receptor occupancies and PK-PD model simulations. *Results* MCLA-128 PK in cynomolgus monkeys was described by a two-compartment model with parallel linear and nonlinear clearance. The xenograft tumor growth model consisted of a tumor compartment with a zero-order growth rate and a first-order dying rate, both affected by MCLA-128. Human doses of 10 to 480 mg q3wk were predicted to show a safety margin of >10-fold compared to the cynomolgus monkey AUC at the no-observed-adverse-effect-level (NOAEL). Doses of ≥360 mg resulted in predicted receptor occupancies above 99% (C_max_ and C_ave)_. These doses showed anti-tumor efficacy in the PK-PD model. *Conclusions* This analysis predicts that a flat dose of 10 to 480 mg q3wk is suitable as starting dose for a First-in-Human study with MCLA-128. Flat doses ≥360 mg q3wk are expected to be efficacious in human, based on receptor occupancies and PK-PD model simulations.

## Introduction

MCLA-128 is a full length humanized IgG1 bispecific monoclonal antibody (mAb) with enhanced antibody-dependent cell mediated cytotoxicity (ADCC) targeting the HER2 and HER3 receptor tyrosine kinases. MCLA-128 is developed to overcome HER3-mediated resistance to EGFR and HER2-targeted therapies. Current HER2-targeted therapies are approved for HER2-amplified breast and gastric cancers, either as single agents or in combination with other anti-cancer drugs [1, 2]. However, a proportion of patients treated with these therapies show primary or acquired resistance [3, 4]. A major resistance mechanism is mediated via HER3 activation. Its ligand heregulin drives dimerization of HER3 with HER2, resulting in potent activation of the PI3K/AKT pathway with subsequent enhanced growth and survival of HER2-amplified tumors. Heregulin stimulation was shown to mediate resistance to trastuzumab and lapatinib therapy [5–7]. Alternatively, HER3 upregulation in HER2-amplified tumors can also result in ligand-independent dimerization of HER3 with HER2 and enhanced cell survival (7). The simultaneous targeting of HER2 and HER3 by MCLA-128 could overcome this resistance. MCLA-128 is expected to directly inhibit tumor growth by blocking HER2:HER3 signaling and, through the ADCC mechanism, eliminate tumor cells via recruitment of natural killer effector cells to tumor cells coated with MCLA-128.

In vitro results show that MCLA-128 inhibits proliferation of HER2 over-expressing and HER2-low cells stimulated with heregulin. MCLA-128 shows significantly higher potency than lapatinib, trastuzumab alone or to the combination of trastuzumab and pertuzumab [8].

Preclinical in vivo research was conducted in cynomolgus monkeys and in tumor xenograft models in mice to understand the preclinical pharmacokinetics (PK) and pharmacodynamics (PD) of MCLA-128. The aim of this study was to develop a preclinical PK-PD model for MCLA-128 based on (i) PK characteristics of MCLA-128 in cynomolgus monkeys and (ii) the effect of MCLA-128 on tumor growth in mouse xenograft models. The preclinical PK model was used to predict the safe starting dose in humans, to support selection of the First-In-Human dose for the Phase I dose-finding trial, and to identify the clinical doses that reach a sufficient percentage of receptor occupancy. In addition, the full preclinical PK-PD model was used to evaluate the anti-tumor activity of the proposed clinical doses.

## Material and methods

### Generation of MCLA-128

MCLA-128 was engineered using proprietary CH3 technology, and is composed of two identical common light chains and two different heavy chains (anti-HER2 and anti-HER3). ADCC-enhancement was achieved by low fucose glycoengineering using the GlymaxX® technology [8].

### Data (1) PK of MCLA-128 in cynomolgus monkeys

PK data from 28 cynomolgus monkeys was combined from a single dose toxicity study and the first week of a repeated dose toxicity study. In the single dose toxicity study, 14 blood samples per animal were drawn and sampling times ranged from 0 to 1007 h. Each dosing regimen of 10 mg/kg, 30 mg/kg and 100 mg/kg was administered intravenously to one female and one male animal (total *n* = 6). In the repeated dose toxicity study, 22 animals received a weekly dose of MCLA-128 for five weeks; only data from the first week was included in the analysis. A dosing regimen of 10 mg/kg (*n* = 6), 30 mg/kg (n = 6) and 100 mg/kg (*n* = 10) was administered with equal distribution between female and male animals. Ten samples per animal were drawn and sampling times ranged from 0 to 168 h. MCLA-128 was quantified in serum using a validated electrochemiluminescence immunoassay (lower limit of quantification (LLOQ): 78 ng/mL). The experiments in cynomolgus monkeys were conducted at Charles River Laboratories Edinburg (preclinical services). All procedures were performed in accordance with the UK Animals (Scientific Procedures) Act, 1986, approved by institutional ethical review committees and conducted under the authority of the Project License.

### Data (2): Antitumor efficacy in xenograft models

MCLA-128 antitumor activity was evaluated in a human breast carcinoma model using the JIMT-1 cell line. In this experiment, 8 to 12 weeks old female CB.17 SCID mice were injected subcutaneously in the right flank with 5·10^6^ JIMT-1 tumor cells. Treatment started 8 days after tumor cell implantation, with tumor volumes ranging from 108 to 172 mm^3^. Animals (*n* = 10 per group) received weekly intraperitoneal (i.p) injections of either phosphate bufferd saline, MCLA-128 at 2.5 mg/kg or MCLA-128 at 25 mg/kg for four weeks (4 doses in total). Mice were euthanized on day 68 or when tumor size reached 800 mm^3^. Tumors from mice were extracted 24 h after the last dose. Tumor size was determined with a caliper twice weekly and tumor volume was calculated using the following equation: tumor volume (mm^3^) = (width^2^·length) · 0.5. Efficacy data were used to develop the PD model.

Mouse xenograft studies were performed by Charles River Discovery Services North Carolina, USA and the experimental protocol was approved by the site’s Institutional Animal Care and Use Committee. The facility is accredited by the Association for Assessment and Accreditation of Laboratory Animal Care International (AAALAC).

### PK modeling

The structural PK characteristics of monoclonal antibodies (mAbs) are usually described by a two-compartment model with either linear, nonlinear or parallel linear and nonlinear clearances [9]. Antibodies follow primarily linear clearance through cellular uptake followed by lysosomal degradation, mediated by the neonatal Fc receptor (FcRn). In addition, the Fab region of the antibody can bind to the target receptor, leading to a saturable clearance pathway, known as target mediated drug disposition (TMDD) [10, 11]. The starting point for model development in the current analysis was a two-compartment model for which different combinations of linear and nonlinear clearance were evaluated. The PK model was directly scaled to a 70 kg human using allometric scaling.

### Tumor growth modeling

Non-perturbed tumor growth models were evaluated in the untreated mice. Different growth models were evaluated, such as Gompertz growth, zero-order growth (linear) and first-order (exponential) growth [12].

PK sampling was not performed in the xenograft study. Therefore, the previously established PK model developed based on cynomolgus monkey data was allometrically scaled to a 0.02 kg mouse to predict concentration-time profiles and assess their relation to tumor growth in the treated animals [13, 14].

The MCLA-128 anti-tumor effect was modeled to impact either the tumor growth rate (K_G_), the tumor dying rate (K_D_) or both. Different models to describe these effects were evaluated, such as direct effect models, indirect response models and use of transit and effect compartments, to establish the correct delay in effect, seen in the individual plots describing tumor volume over time. The drug effect was modeled as either a linear effect or an E_max_ model. Additionally, a tumor growth rate increase over time was considered.

### Statistical model development

Inclusion of inter individual variability was considered for all structural model parameters as follows:

$$ {P}_i={P}_{pop}\cdot \exp \left({\eta}_i\right) $$

Where *P*_*i*_ is the individual parameter estimate for individual *i*, and *P*_*pop*_ is the typical population parameter estimate, and where *η*_*i*_ was assumed to be distributed normally distributed with mean 0 and variance ω^2^. Residual unexplained variability was described as a proportional and additive error model for the PK model:

$$ {C}_{obs, ij}={C}_{pred, ij}\cdot \left(1+{\varepsilon}_{p, ij}\right)+{\varepsilon}_{a, ij} $$

For the PD part of the model residual variability was described by a proportional error model:

$$ {C}_{obs, ij}={C}_{pred, ij}\cdot \left(1+{\varepsilon}_{p, ij}\right) $$

Where *C*_*obs*, *ij*_ represents the observed concentration for individual *i* and observation *j*, *C*_*pred*, *ij*_ represents the individual predicted concentration, *ε*_*p*, *ij*_ the proportional error and *ε*_*a*, *ij*_ the additive error, both distributed following N (0,σ^2^).

For PK data, the first data point below the LLOQ (78 ng/mL) was fixed to LLOQ/2 and a fixed additive error component of LLOQ/2 was included in the model to account for uncertainty in these observations [15].

### Model evaluation

Models were evaluated based on general goodness-of-fit (GOF) plots, plausibility, stability and precision of parameter estimates and change in objection function value (OFV) where a *p* < 0.01 was considered significant, meaning that a OFV drop of >6.63 (degree of freedom = 1) was considered as a significant improvement.

### Software

Data management, graphical evaluation and simulations were performed using R (version 3.0.1) [16]. Nonlinear mixed effects modeling was performed using NONMEM (version 7.3.0, ICON Development Solutions, Ellicott City, MD, USA) and Perl-speaks-NONMEM (version 4.4.8) [17, 18]. Pirana (version 2.9.2) was used as graphical user interface [19]. All models were estimated using First Order Conditional Estimation method with η-ε interaction (FOCE-I).

### Determination of the safe starting dose and clinical efficacious dose

A safe starting dose for the First-In-Human study of MCLA-128 was identified by calculation of safety margins based on the simulated exposure in humans at different dose levels. Subsequently, a clinical target exposure and dose was determined by calculation of receptor occupancies for different dose levels, based on the simulated exposures in human and the estimated K_m_ value. Doses with a receptor occupancy above 99%, based on the maximum and average MCLA-128 concentration in the first cycle, were expected to have a clinical effect. In addition, a simulation with the tumor growth model was performed in mice, to evaluate the potential human anti-tumor efficacy of the proposed clinical dose regimens.

First, the safety margins were calculated for different simulated dose levels. The safety margins were based on the no-observed-adverse-effect-level (NOAEL) of MCLA-128 in monkeys included in the multiple dose toxicity study, which was determined at 100 mg/kg. The mean AUC_0-inf_ of 193 g∙hr/L was calculated using the PK data of the monkeys included in the single dose toxicity study that received 100 mg/kg, to assure that the exposure to MCLA-128 was not compromised by possible generation of anti-drug antibodies. The safety margin was calculated by dividing the 193 g∙hr/L AUC_0-inf_ by the predicted model-based AUC_0-inf_. The AUCs were computed using a non-compartmental analysis of both the observed and simulated data. Second, the receptor occupancies based on the maximal, trough and average concentrations (C_max_ C_trough_ and C_ave_, respectively) were calculated, using the same simulated exposure data as used for obtaining the safety margins. The receptor occupancies were calculated based on the estimated K_m_ value, using the following equation:

$$ \% RO=100\cdot \frac{C_{\mathit{\max}\  or\ trough\ or\ average}}{K_m+{C}_{\max\ or\ trough\ or\ average}} $$

Lastly, to evaluate the potential human anti-tumor efficacy the proposed clinical dose regimens for MCLA-128 were evaluated with the preclinical PK-PD model in mice. Tumor stasis at day 21 was evaluated after applying a regimen of a weekly dose for three weeks. The dose input was chosen so that the total exposure (AUC) of the three doses, mimicked the exposure of proposed clinical doses administered once in a 21-day cycle.

## Results

### PK model

A two-compartment model with parallel linear and nonlinear clearances from the central compartment described the data best. The nonlinear clearance was described using Michaelis-Menten kinetics. The final model structure was defined by the following differential equations:

1 $$ \frac{d\left({A}_1\right)}{d(t)}=-\frac{CL}{V_1}\cdot {A}_1-\frac{V_{max}\cdot {C}_1}{K_m+{C}_1}-\frac{Q}{V_1}\cdot {A}_1+\frac{Q}{V_2}\cdot {A}_2\kern1em $$

2 $$ \frac{d\left({A}_2\right)}{d(t)}=\frac{Q}{V_1}\cdot {A}_1-\frac{Q}{V_2}\cdot {A}_2\kern1em $$

Where CL represents the linear clearance, Q the intercompartmental clearance, V_1_ the volume of distribution in the central compartment and V_2_ the volume of distribution in the effect compartment, A_1_ the amount of drug in the central compartment, V_max_ the maximum elimination rate, C_1_ the drug concentration in the central compartment, K_m_ the drug concentration at which half the drug-targets are occupied and A_2_ the amount in the peripheral compartment. Scaling of the model to a 70 kg human or 0.02 kg mouse was performed using the following equations, respectively:

$$ {\displaystyle \begin{array}{l}{P}_{monkey\_ pop}={\theta}_{human\_ pop}\cdot {\left(\frac{WT}{70}\right)}^{factor}\\ {}{P}_{mouse\_ pop}={\theta}_{human\_ pop}\cdot {\left(\frac{0.02}{70}\right)}^{factor}\end{array}} $$

**Fig. 1:**
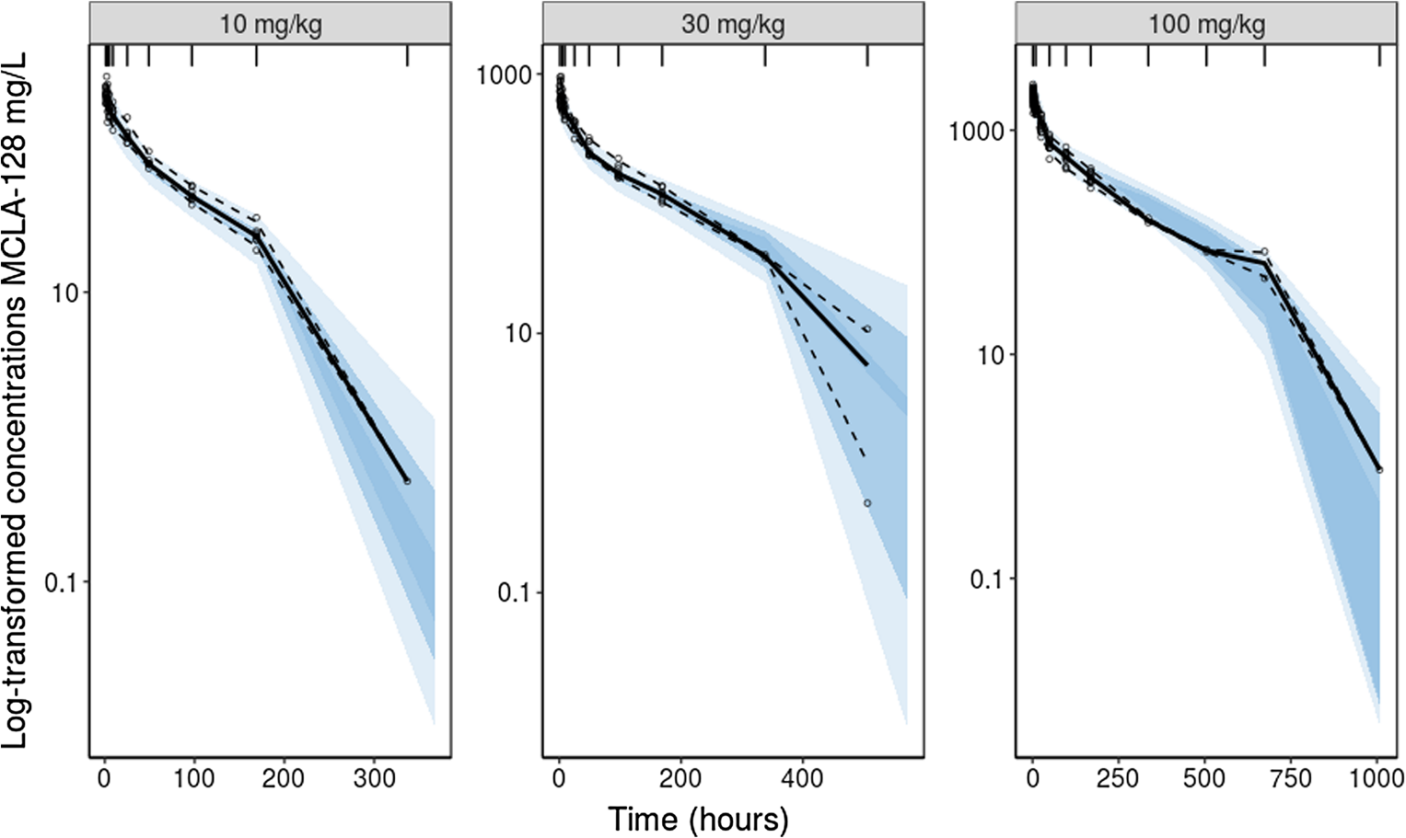
Visual Predictive Checks (VPCs) for PK data from cynomolgus monkeys stratified on dose group (10 mg/kg, 30 mg/kg, 100 mg/kg). The solid line represents the median of the observed MCLA-128 concentrations, the dashed lines represents the 5^th^ and the 95^th^ percentiles of the observed data. The shaded areas show the 95% confidence interval of the simulated data for the corresponding percentiles (*n* = 500).

On CL, Q, and V_max_ a factor of 0.75 was used and on V_1_ and V_2_ a factor of 1 was used. MCLA-128 is fully cross-reactive with cynomolgus monkey HER2 and HER3 receptors and mice were implanted with human HER2 and HER3 expressing tumors. Therefore, K_m_ was not scaled and fixed to the parameter estimate in cynomolgus monkeys (0.219 mg/L) for both human and mice [20, 21]. Parameter estimates for a 70 kg human and the calculated scaled parameters for a 0.02 kg mouse are depicted in Table 1. The visual predictive checks (VPCs) demonstrate that the model accurately describes the observed PK data in cynomolgus monkeys for each dose-group (Fig. 1).

**Table 1:** Allometrically scaled PK parameters describing MCLA-128 concentration-time data in cynomolgus monkeys and mice. Parameters estimated for a 70 kg humand and scaled to 0.02 kg mice.

	Units	Estimates	RSE (%)	Shrinkage (%)	Scaled parameters mice
Parameter					
CL	L/h	0.0125	9.4	–	2.75∙10^−5^
V_1_	L	3.17	2.9	–	9.06∙10^−4^
Q	L/h	0.0313	6.5	–	6.88∙10^−5^
V_2_	L	3.51	14.7	–	1.00∙10^−3^
V_max_	mg/h	0.500	10.3	–	1.10∙10^−3^
K_m_	mg/L	0.219	Fixed	–	0.219
Between-subject variability (%)					
CL	CV	13.2		12.6	–
V_1_	CV	14.6		3.8	–
Residual variability					
Prop	SD	0.108	1	10.6	–
Add	SD	0.039	Fixed		–

CL, linear clearance; V_1_, volume of distribution in the central compartment; Q, distributional clearance; V_2_, volume of distribution in the peripheral compartment; V_max_, maximum velocity, when all drug-targets are saturated; K_m_, concentration at which half the drug-targets are occupied; Prop, proportional error; Add, additive error; CV, coefficient of variation; SD, standard deviation

### PK-PD model

**Fig. 2:**
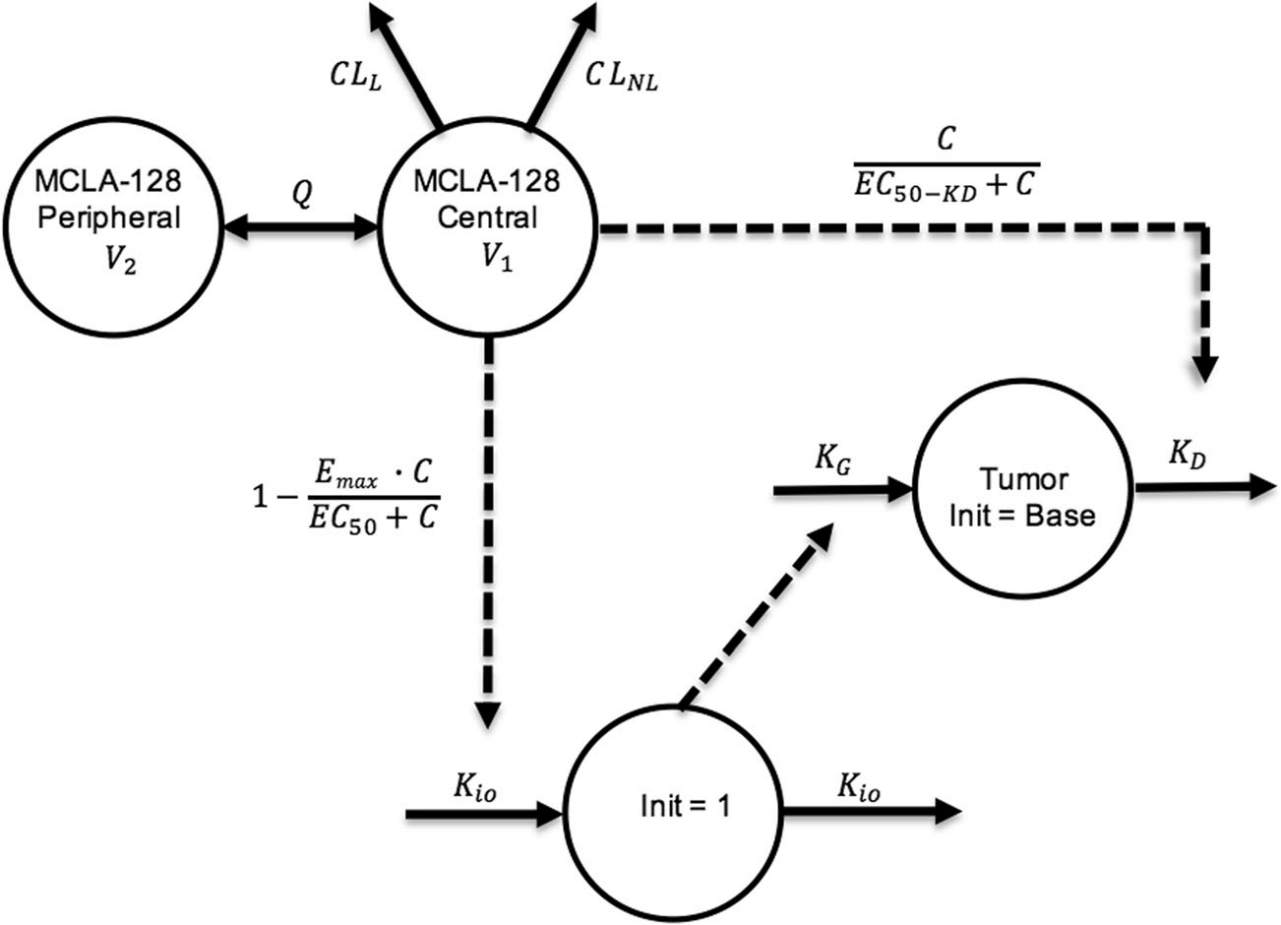
Schematic structure of the PK-PD model in mice. CL_L_ = linear clearance, CL_NL_ = nonlinear clearance, V_1_ = volume of distribution central compartment, V_2_ = volume of distribution peripheral compartment. K_G_ = zero-order tumor growth rate, K_D_ = tumor dying rate, K_io_ = production and loss of drug effect on K_G_, E_max_ = maximum effect of MCLA-128 on K_io_ (fixed to 1). EC_50_ = MCLA-128 concentration with 50% of maximum effect on K_io_, EC_50_KD_ = concentration MCLA-128 with 50% of maximum effect on K_D._ Dotted lines = drug effects

**Fig. 3:**
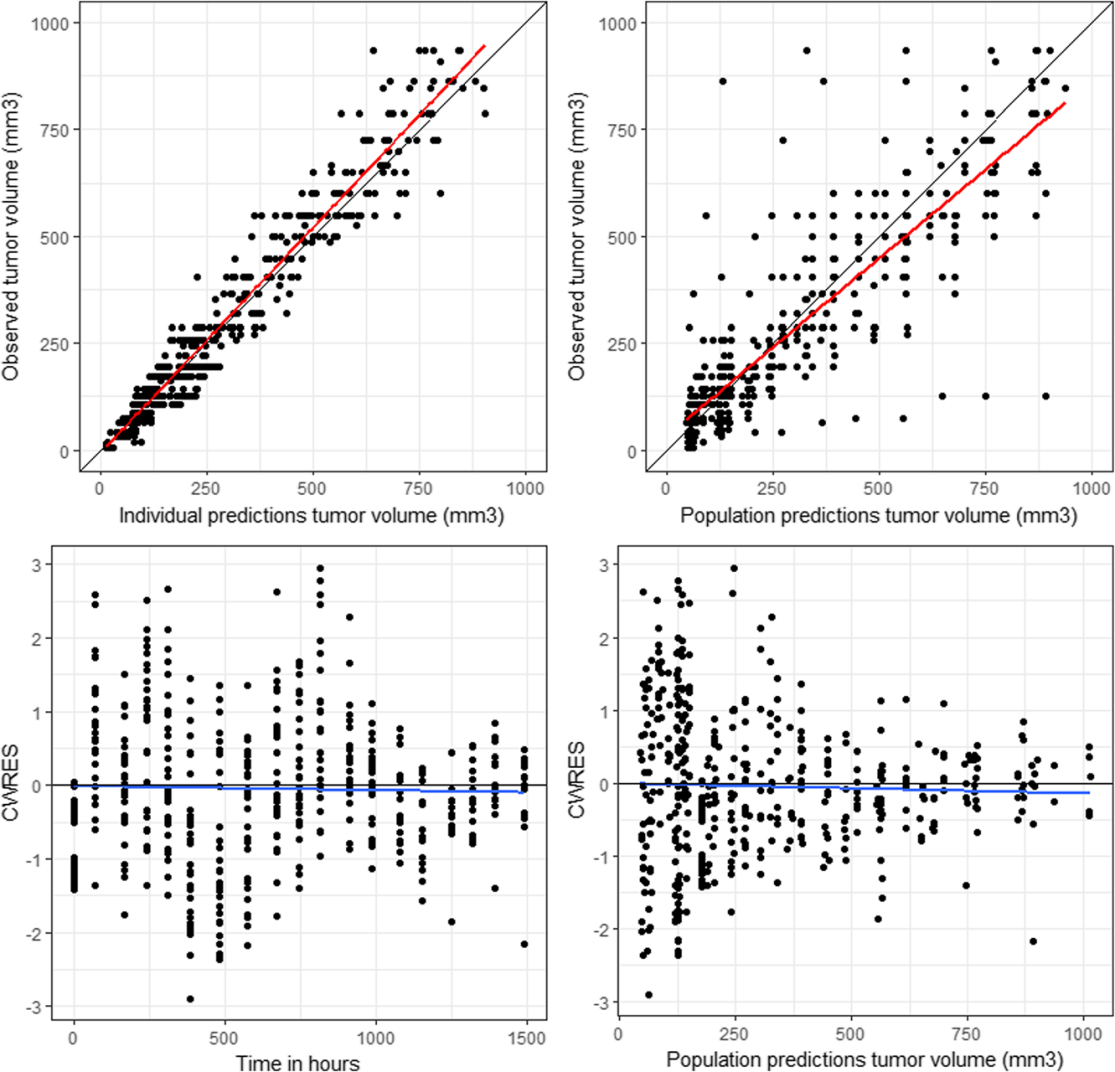
Goodness of fit plots tumor growth model, CWRES = conditional weighted residuals

**Fig. 4:**
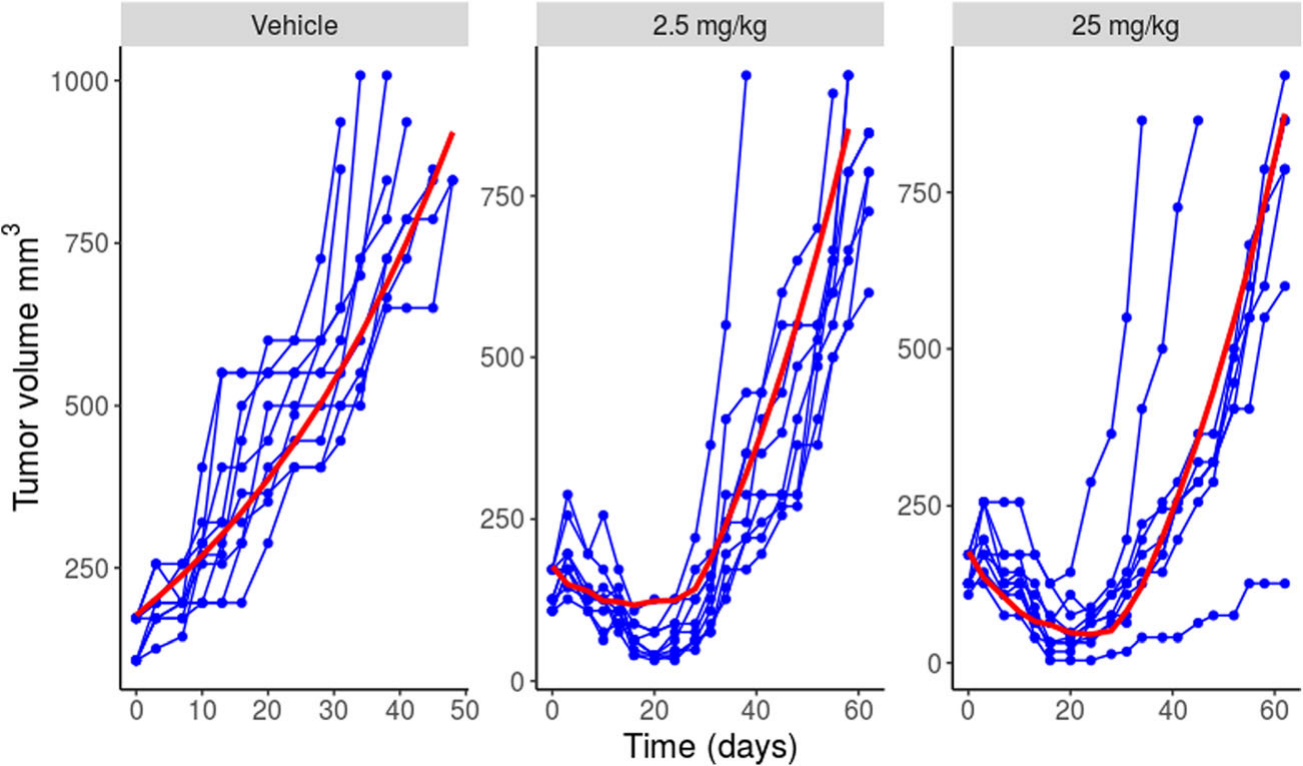
Individual tumor volume over time curves for each dose group (vehicle, 2.5 mg/kg and 25 mg/kg). Blue dots and lines = observed tumor volumes, red line = population prediction

The preclinical PK-PD model was based on the scaled PK model from cynomolgus monkeys to mice and the xenograft experiments conducted in mice. First, the non-perturbed tumor growth in the vehicle-treated mice was modelled. This was best described by a zero-order growth rate (K_G_). The MCLA-128 anti-tumor effect in this experiment was modeled to target the proliferation and dying rate (K_G_ and K_D_) of the tumor. The effect on K_G_ was described by an indirect response model, where the in-rate (K_io_) in the indirect effect compartment was affected by the predicted MCLA-128 concentration using an inhibitive E_max_ equation. The effect of the predicted MCLA-128 concentrations on K_D_ was modeled directly with an E_max_ model. An indirect response model and effect compartment model were evaluated to investigate a delay of the effect on K_D_, but this could not be identified. Addition of an increasing tumor growth rate over time (modelled by inclusion of the λ term) led to a significant increase of model fit and was implemented in the final model. The structural model is depicted in Fig. 2 and the estimated PD model parameters are depicted in Table 2. Goodness of fit plots and a plot demonstrating observed tumor volume versus predicted population mean, demonstrated adequate fit of the model (Figs. 3 and 4) .

**Table 2:** Population parameter estimates for the preclinical PK/PD model: the effect of MCLA-128 on tumor growth in JIMT-1 xenograft models

Parameter	Units	Parameter estimates	RSE (%)	Shrinkage (%)
Population PD parameters in mice				
Tumor baseline value (Base)	mm^3^	177	6.7	–
Zero order tumor growth rate (K_G_)	hr^−1^	0.338	22.2	–
First order tumor dying rate (K_D_)	mm^3^/h	0.004	15.9	–
Production and loss of drug effect on K_G_ (K_io_)	hr^−1^	0.143	18.3	–
MCLA-128 concentration with 50% of maximum effect on K_io_ (EC_50_)	mg/L	2.60	47.7	–
MCLA-128 concentration with 50% of maximum effect on K_D_ (EC_50_KD_)	μg/L	0.0102	25.1	–
Progression factor	week^−1^	0.172	23	–
Between-subject variability (%)				
Baseline	CV	20.6		16.8
K_G_	CV	55.1		1.10
K_D_	CV	35.5		24.8
Residual variability				
Proportional residual error tumor compartment	CV	25.6	7.4	5.9

Population PK parameters were scaled to mice to drive the tumor growth model, parameters reported in Table 1

RSE, relative standard error; CV, coefficient of variation; SD, standard deviation

### Safe starting dose and clinical efficacious dose

**Fig. 5:**
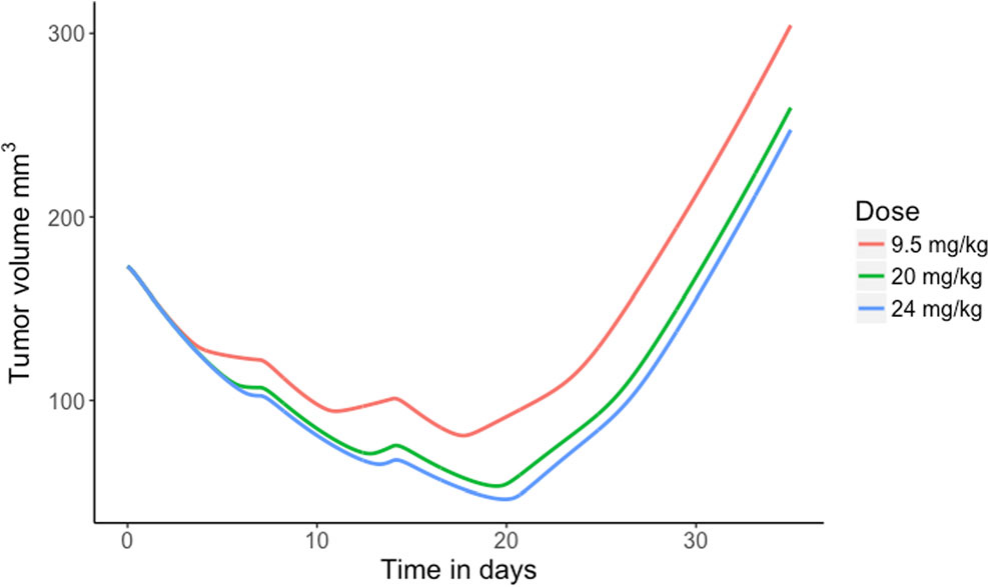
Simulation of tumor growth in mice, with administered doses of 9.5, 20 and 24 mg/kg q1wk for three weeks, corresponding with AUCs after 360, 750 and 900 mg flat dose of MCLA-128 given q3wk to humans

The safety margins and percentages receptor occupancies at predicted maximum, trough and average concentrations were calculated for the anticipated clinical doses. Results are depicted in Table 3. Clinical doses ranging from 10 to 480 mg flat dose MCLA-128 showed a safety margin >10-fold and doses ≥360 mg had an expected receptor occupancy higher than 99% for both C_max_ and C_ave_. In addition, a sensitivity analysis for the K_m_ was conducted for K_m_ values ranging from 0.0219 mg/L to 2.19 mg/L, since this parameter showed a high relative standard error (RSE) of 74% value in the monkey estimation. The sensitivity analysis showed permanent adequate receptor occupancies for varying K_m_ values and values of higher than 99% for doses starting from 160 mg (K_m_ of 0.0219 mg/L) and 750 mg (K_m_ 2.19 mg/L). Subsequently, the tumor volumes over time in 0.02 kg mice were simulated with the established preclinical PK-PD model for dose levels of 9.5 mg/kg, 20 mg/kg and 24 mg/kg given once every week (q1wk) for 3 weeks (Fig. 5). These dose levels had AUCs corresponding to the 360 mg, 750 mg and 900 mg flat dose of MCLA-128 given q3wk in the First-In-Human study, and demonstrated profound tumor stasis at day 21.

**Table 3:** Simulation results of MCLA-128 exposure (AUC) and predicted receptor occupancy (RO) for different flat doses of MCLA-128 administered to humans once every 3 weeks

Flat dose (mg)	AUC (g∙hr/L)	Safety Margin	C_max_ %RO	C_ave_ %RO	C_trough_ %RO
10	0.031	6226	93.5	22.6	0.021
20	0.10	1930	96.6	48.8	0.074
40	0.33	585	98.3	75.1	0.253
80	1.00	193	99.1	90.2	0.874
160	2.97	65	99.6	96.4	3.29
240	5.57	35	99.7	98.1	8.12
360	10.4	19	99.8	99.0	23.8
480	16.0	12	99.9	99.3	75.7
600	22.4	9	99.9	99.5	96.5
750	31.4	6	99.9	99.6	98.4
900	41.1	5	99.9	99.7	99.0
1000	47.9	4	99.9	99.7	99.2
1200	62.0	3	99.9	99.8	99.3

C_max_, maximum concentration; C_ave_, average concentration; RO, receptor occupancy; %RO = 100 · C_max or trough or average_ / (K_m_ + C_max or trough or average_)

## Discussion

In this analysis, the preclinical PK characteristics of MCLA-128 were quantified in cynomolgus monkeys and subsequently predicted for humans. The PK profiles were well described by a two-compartment model with parallel linear and nonlinear clearance pathways. Predicted parameters were in accordance with previously published PK characteristics of different therapeutic mAbs in human, with a median (range) of V_1_ and V_2_ of 3.1 L (2.4–5.5) and 2.8 L (1.3–6.8), respectively, and for linear clearance (CL_L_) 0.013 L/h (0.003–0.223) [9]. Estimates for V_max_ and K_m_ varied widely among the different IgG mAbs, but the Michaelis Menten estimates for MCLA-128 were within this wide range [9]. Cynomolgus monkeys are considered to be the most relevant species to predict PK of monoclonal antibodies in human [22]. In addition, healthy cynomolgus monkeys express HER2 and HER3 receptors with binding epitopes for MCLA-128 that are conserved between human and cynomolgus monkeys. This is a requirement to determine the nonlinear (target mediated) clearance pathway. Moreover, the design of MCLA-128 using the CH3 engineering and the low fucose glycoengineering technologies did not alter the IgG PK characteristics of the compound, since PK parameters were in the range of previously published parameters of other therapeutic IgG mAbs.

Subsequently, the established PK model was used to predict safety in humans. Dose levels of 10 to 480 mg flat dose of MCLA-128 given q3wk have predicted AUCs that are at least 10-fold lower than the NOAEL corresponding AUC in monkeys. Doses of 10 to 480 mg were, therefore, considered suitable as a First-In-Human starting dose. However, following the CHMP guideline on identifying and mitigating risk for such studies [23], other non-clinical safety pharmacology and toxicology data should also be taken into account to determine the optimal starting dose for the Phase I dose-escalation trial, including the identification of the factors of risk. Concerns may be derived from particular knowledge or lack thereof regarding the mode of action, the nature of the target, and/or the relevance of animal models. Obtaining the exposures in humans using a PK modeling and simulation approach is preferred over traditional calculation of the human equivalent dose, since (non-linear) pharmacokinetic characteristics are taken into account [24, 25]. In addition, antibodies are suitable compounds for this approach, since no metabolites are formed and no enzymatic metabolism is present, which might trouble the prediction of exposure from animal to human [9, 22].

The PK model was then used to determine the pharmacological active doses based on receptor occupancies. Doses ≥360 mg flat dose of MCLA-128 given q3wk are expected to reach a receptor occupancy superior to 99% at C_max_ and C_avg_, and at C_trough_, of 24%. However, the receptor occupancies are calculated using the model estimated K_m_ value based on healthy cynomolgus monkeys. These cynomolgus monkeys did not bear HER2/HER3 expressing tumors, but only endogenously expressed HER3 and HER2 epitopes. It is expected that tumor-bearing patients demonstrate higher expression of HER2 and HER3 receptors. Therefore, the clinical model estimate for the V_max_ and K_m_ could be different. The sensitivity analysis demonstrated that for a 10-fold increase in the K_m_ value, a 750 mg flat dose MCLA-128 would attain a receptor occupancy of 99% at C_max_. In addition, for trastuzumab, a K_m_ value of 3.7 mg/L has been identified in patients with HER2-amplified advanced gastric or gastroesophageal junction cancer [26]. This K_m_ is in the same order of magnitude as the K_m_ of MCLA-128 used in the sensitivity analysis (2.19 mg/L). Moreover, in breast cancer, only linear PK models for trastuzumab have been identified potentially indicating that all target is saturated and, that the target mediated clearance of trastuzumab is of minor importance in breast cancer at therapeutic dose levels [27].

Finally, the proposed effective doses in human were evaluated using the preclinical PK-PD model. The final tumor growth (PD) model included an effect on the tumor growth rate and on the tumor dying rate. The low EC_50_ value for the effect on K_D_ (EC_50_KD_) suggests that the anti-tumor activity is present during almost the complete time course between administrations in mice for doses of 2.5 mg/kg and higher. The JIMT-1 cell line has higher HER2 expression than HER3 expression and is reported to overexpress the HER3 ligand heregulin [28, 29]. The JIMT-1 cell line is resistant to HER2 targeted therapies and partially dependent on autologous heregulin for growth which can be effectively blocked in vitro by MCLA-128 [28]. The JIMT-1 cell line was therefore considered suitable for determining the direct effect of MCLA-128 on tumor growth in a xenograft setting. However, this approach may underpredict the true anti-tumor efficacy of MCLA-128 due to the inherent limitations of the xenograft models: immunodeficient mice were used in the JIMT-1 xenograft model and therefore the ADCC-related mechanism of action could not be evaluated. As a result, the low EC_50_KD_ is expected to represent the natural dying rate of the tumor resulting from a decrease in tumor growth rate. On the other hand, the exposure of MCLA-128 in mice was predicted using an allometrically scaled PK model, the true exposure in mice is expected to be higher, since humanized mAbs have a high affinity for mouse and rat FcRn, resulting in a decrease in linear clearance, subsequently resulting in higher concentrations [30]. Therefore, it is expected that the EC_50_ parameters for anti-tumor activity are higher than estimated in the preclinical PK/PD model. It is unclear how these two findings are balanced, hence how they affect the preclinical predictions of tumor growth. However, we expect that the true anti-tumor efficacy is stronger than simulated, since lack of the ADCC effect is expected to have a stronger impact on predictions than an increase in the EC_50_ parameter. Nevertheless, MCLA-128 demonstrated a profound anti-tumor activity in mice.

Receptor occupancies based on C_max_ and C_ave_ were expected to be >99% starting as of 360 mg MCLA-128 given q3wk and receptor occupancies based on C_trough_ at the end of a 3-week dosing interval were > 99% as of 900 mg (Table 3). Since the anti-tumor effects of MCLA-128 are mediated via receptor binding, it is expected that a further increase in dose will not lead to a significant increase in effect, for doses reaching receptor occupancies >99%. Likewise, both drug effects in the PK-PD model were described by an E_max_ model, confirming an asymptotic approach of the maximum effect. However, the tumor growth model was not able to capture a plateau in effect starting from approximately 360 mg or 900 mg q3wk, because data about receptor and receptor-drug complex concentrations was lacking.

In general, in this analysis all available relevant PK and PD data before start of the First-in-Human trial were combined in a comprehensive modeling framework to fully evaluate the safe starting dose and predicted efficacious dose range. This framework can be applied similarly for the evaluation of other monoclonal antibodies.

## Conclusion

A preclinical predictive PK-PD model describing the relation between MCLA-128 exposure and tumor volume over time was developed and demonstrated the anti-tumor efficacy of MCLA-128. The calculation of the safety margins demonstrated that flat doses of 10 to 480 mg MCLA-128 given q3wk are expected to be safe as starting dose for a First-In-Human study with MCLA-128 based on the NOAEL exposure in cynomolgus monkeys. However, other non-clinical safety pharmacology and toxicology data should also be taken into consideration to determine the optimal starting dose for the Phase I dose escalation trial, including the identification of the factors of risk. The simulations and the estimations of receptor occupancy for different dose levels showed that flat doses ≥360 mg of MCLA-128 given q3wk are likely to be efficacious in human.
